# Prevalence and Morphology of C-Shaped Canals: A CBCT Analysis in a Korean Population

**DOI:** 10.1155/2021/9152004

**Published:** 2021-05-29

**Authors:** Sung Eun Yang, Tae Yeon Lee, Kyung Jae Kim

**Affiliations:** ^1^Department of Conservative Dentistry, Seoul St. Mary's Dental Hospital, College of Medicine, The Catholic University of Korea, Seoul, Republic of Korea; ^2^Department of Conservative Dentistry, Yeouido St. Mary's Hospital, College of Medicine, The Catholic University of Korea, Seoul, Republic of Korea; ^3^Private Dental Clinic, Seoul, Republic of Korea

## Abstract

This retrospective study of roots with C-shaped canals investigated their prevalence, configuration type, and lingual wall thickness, as well as the panoramic radiographic features of roots in permanent mandibular second molars confirmed to have C-shaped canals on cone-beam computed tomography (CBCT) in a Korean population. In total, 1884 CBCT images of mandibular second molars were examined by two endodontists to analyze the presence of C-shaped canals according to age and sex. The bilateral occurrence of C-shaped roots and their morphology on panoramic radiography were assessed and statistically analyzed using the chi-square test. The classification of Fan et al. was applied to categorize the configurations of C-shaped canals. The lingual wall thickness was calculated in the mesial, middle, and distal areas at the orifice and at 5 mm from the apex. The Mann–Whitney *U* test was used to analyze the mean difference of lingual wall thickness between the apex and orifice level. A *P* value of 0.05 was considered to indicate statistical significance in the statistical analyses. Of 2508 mandibular second molars, 924 (36.8%) had C-shaped root canals. The prevalence was significantly lower in the over 61 age group (24.08%) than in the 21–30-year age group (40.02%) and was higher in women (42.32%). Most cases were bilateral (85.9%). The C1 type was the most common (35.3%). The prevalence of C1 type canals decreased, while that of C3b type canals increased with age. In 75.2% of teeth having C-shaped root canals on CBCT, fused roots were observed on panoramic views. The difference in the lingual wall thickness at the orifice and 5 mm from the apex was significant in the middle area in all configurations of C-shaped root canals. When performing nonsurgical or surgical endodontic procedures of the mandibular second molars, clinicians should consider age, sex, ethnicity, and anatomical variations.

## 1. Introduction

Success of root canal treatment requires a thorough understanding of variations in canal configuration [1]. The C-shaped canal is an important example of such a variation and was first described using this term in 1979 by Cooke and Cox [2]. The main cause of this anatomical variant, in which a continuous slit or web forms a connection between individual root canals, may be the failure of Hertwig's epithelial root sheath to fuse to the buccal or lingual root surface [3]. C-shaped canals are known to appear most frequently in the mandibular second molars [4].

C-shaped canals show a varying prevalence across populations. In studies of Caucasian populations, their prevalence was reported to be 2.7–7.6% [2, 4], and in the study of middle east populations [5], their prevalence was reported to be 7.9% while higher prevalence rates were reported in northeast Asia, with rates of 31.5% in Chinese [6] and 32.7% in Koreans [7].

The presence of a thin wall and a narrow isthmus in C-shaped canals poses a risk for strip perforation [8]. Therefore, clinicians have difficulty fully understanding the complex anatomy of C-shaped canal. For this reason, clinicians should understand the configuration of C-shaped canals and take precautions to prevent instrumentation errors for success of root canal treatment and thorough understanding of the internal anatomy of root canals, including canal wall thickness [9].

Cone-beam computed tomography (CBCT) has been suggested as a useful method for studying root canal anatomy, since it provides three-dimensional images that allow a more accurate and detailed knowledge of the root canal system than is possible using conventional two-dimensional periapical radiographs [10–13]. The noninvasive nature of CBCT also enables studies with larger sample sizes than was possible in previous research using microscopy [10–13].

This retrospective study investigated C-shaped canals found on CBCT in permanent mandibular second molars in a Korean population, with a focus on their prevalence, configuration, type, and lingual wall thickness. Furthermore, an attempt was made to identify root forms on panoramic radiography that could predict C-shaped root anatomy as confirmed by CBCT.

## 2. Materials and Methods

### 2.1. Ethical Considerations

The study received approval from the Institutional Review Board of Seoul St. Mary's Hospital, Catholic University of Korea (KC16EISI0332).

### 2.2. Data Collection Procedure

The CBCT images of mandibular molars analyzed in this study were obtained from clinical examinations conducted at Seoul St. Mary's Dental Hospital from June 2017 to August 2018. CBCT images from 1950 patients were initially assessed, and the scans from 1884 patients (894 men and 990 women; age range: 18-92 years, mean age: 44.7 years; overall, 44.8 years for men and 44.7 years for women) satisfied the following inclusion criteria.

1. Korean patients over 18 years old
2. Images containing mandibular second molars with completely mature apex
3. Images containing mandibular second molars that had neither root filling nor post crown restoration

As 630 patients had a single mandibular second molar and 1254 patients had both mandibular second molars, 2508 lower mandibular second molars were analyzed. The study included 973 patients for whom both panoramic radiographs and CBCT scans were available.

### 2.3. Radiographic Evaluation

The CBCT images were obtained with a Q-face device (HDX Corp., Seoul, Korea) using a scan time of 24 seconds at 85 kV and 8 mA with a 160 × 80 mm^3^ voxel size and 0.20 mm slice. In the axial CT images, the mandibular second molars were evaluated from the pulpal floor to the apex to determine the canal shape. In a calibration step (for both intraobserver and interobserver calibrations), two endodontists examined 100 CBCT images. The evaluators reviewed the images twice, with a 10-day interval between assessments. Reliability was analyzed using kappa statistics, which yielded values of 0.81 (intraobserver) and 0.74 (interobserver). The analysis was conducted using vivo5 software (version 5.3; Anatomage, San Jose, CA, USA). After intraobserver and interobserver calibrations, 1880 CBCT images were selected and assessed independently by the evaluators. Any discrepancies were resolved by consensus based on a discussion.

**Figure 1:**
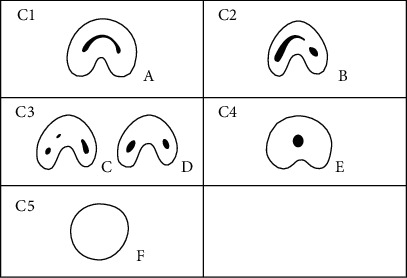
Classification of C-shaped canal configurations by Fan B.

**Figure 2:**
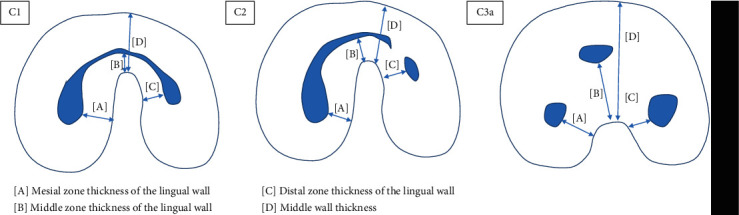
Measurement locations of the C1, C2, and C3a types.

The prevalence of C-shaped root canals according to age, sex, and bilateral occurrence was calculated. The orifice level was defined as 2 mm below the cementoenamel junction in C-shaped roots [14]. Melton's classification as modified by Fan et al. [14] was used to categorize the canal configurations of C-shaped roots at the orifice level according to age (Figure 1). If a panoramic radiograph was available for patients with a C-shaped canal on CBCT, the presence of root separation on the panoramic radiograph was assessed. Lingual wall thickness was measured 5 mm from the apex level and at the orifice and was calculated as the shortest distance from the outer surface of the canal to the outer surface of the lingual wall in each of the mesial, middle, and distal zones (Figure 2 [A], [B], and [C]). These measurements were not made for C4-type canals due to the infeasibility of the zoning system, and the middle lingual wall thickness was not measured in C3b-type canals due to the absence of a root canal in the middle area. The middle wall thickness was defined as the shortest distance from the outer root surface to the lingual surface of the deepest site of a C-shaped root (Figure 2 [D]). The distance measurements were made directly on the films using PACX program (INFINITT health care, Seoul, Korea) with an accuracy of 0.01 mm.

### 2.4. Statistical Analysis

R language version 3.3.3 (R Foundation for Statistical Computing, Vienna, Austria) and T&F version 3.0 (YooJin BioSoft, Goyang, Korea) were used for all statistical analyses. Descriptive statistics were presented as numbers and percentage. The two-sample proportion test was used to compare the sample ratios between age subgroups or C-subtypes. The chi-square test was used to analyze the distribution of sample ratios among C-subtypes in the data from panoramic radiography. The Mann–Whitney *U* test was used to analyze the mean difference in the lingual wall thickness between the orifice and apex according to the configuration. A *P* value < 0.05 was considered to indicate statistical significance in the statistical analyses.

## 3. Results

### 3.1. Prevalence of C-Shaped Canals according to Age, Gender, and Tooth Position

The prevalence of C-shaped canals by age, gender, and tooth position is summarized in Table 1. Of the 2508 lower mandibular second molars observed, 924 (36.8%) had C-shaped canals. C-shaped canals were significantly less prevalent in the over 61 age group (24.08%) than in the 21-30-year age group (40.02%), and the prevalence was lower in the 51-60-age group (29.8%) and over 61 age group (24.08) than in other age groups. The prevalence of teeth with C-shaped root canals was higher in women (42.32%) than in men (29.42%). There was no significant difference in prevalence according to the tooth position.

### 3.2. Unilateral and Bilateral Occurrence of C-Shaped Canals

The prevalence of bilateral (75.3%) and unilateral (24.7%) C-shaped canals by gender is shown in Table 2. In both genders, most C-shaped canals were observed bilaterally, and there was no significant difference according to tooth position.

### 3.3. Configuration of C-Shaped Canals according to Age

**Figure 3:**
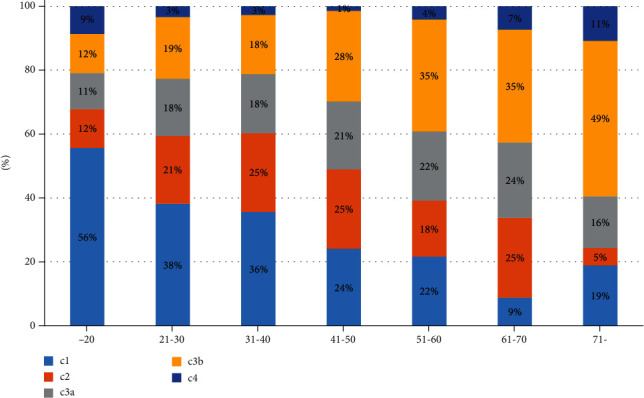
Distribution of configuration types according to age.

In 98.9% (914 teeth) of the teeth identified as having C-shaped root canals (924 teeth), the configuration type was classified at the orifice level as proposed by Fan et al.  Table 3 shows the distribution of C-shaped canals according to type and age. C1 was the most common type (35.3%), while C2 (21.2%) and C3b (21.6%) showed similar proportions. The proportion of C1 canals was significantly higher in those under the age of 30 than in other age groups, and the proportion of C3b canals was significantly higher in the over 61 age group than in those under the age of 60. The prevalence of C1 canals decreased with age, whereas the prevalence of C3b canals increased with age (Figure 3).

### 3.4. Panoramic Radiographic Observations of the Root Shape of Teeth with C-Shaped Canals on CBCT

**Figure 4:**
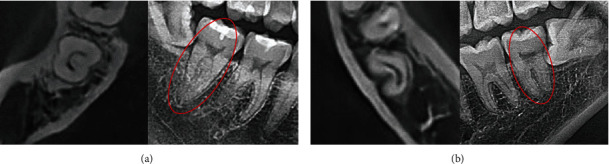
Cone-beam computed tomography (CBCT) scans and panoramic views of teeth observed to have C-shaped canals on CBCT. (a) A case of a mandibular second molar with a fused root on a panoramic radiograph. (b) A case of a mandibular second molar with a separated root on a panoramic radiograph.

Cone-beam computed tomography (CBCT) scans and panoramic views of teeth observed to have C-shaped canals on CBCT were presented in Figure 4. Of the teeth that showed C-shaped canals on CBCT, 242 (24.8%) teeth demonstrated separated roots on panoramic radiographs. The mean middle wall thickness of the teeth with separated roots was 2.98 mm. In 731 teeth (75.2%), fused roots were observed on panoramic radiographs. In these cases, except for 43 teeth with C4 type, the mean middle wall thickness was 4.15 mm. The distribution of configurations of C-shaped root canals observed as fused roots or separated roots on panoramic radiographs is presented in Table 4. There was no difference in the proportion of configuration among the separated roots based on the panoramic view evaluation, whereas in the fused roots on panoramic views, C1 canals (38.2%) were significantly more common than C3a canals (16.6%).

### 3.5. Morphological Analysis of Lingual Wall Thickness Measurements according to the Canal Configuration

Table 5 presents the mean thickness of the lingual wall at the orifice and 5 mm from the apex level in the mesial, middle, and distal zones. In all configuration types, the lingual wall was thinner at 5 mm at the apex than at the orifice level. In all configuration types, the thickness of the middle zone of the lingual wall was significantly different between the orifice and 5 mm from the apex, and the C1 type showed a significant difference in the distal zone of lingual wall thickness between the orifice level and 5 mm from the apex. The lingual wall was thinnest (0.7 mm) in the middle zone at 5 mm from the apex level in the C1 and C3a types.

## 4. Discussion

The C-shaped canal is more common in the Korean population than in other populations, with its reported prevalence ranging from 31% [7] to 44.5% [8]. In the current study, consistent with previous studies involving Koreans, C-shaped canals were observed in 36.8% of mandibular second molars. Differences between populations in the prevalence of C-shaped root canal systems emphasize the influence of ethnicity on the root canal anatomy of mandibular second molars.

C-shaped root canals were significantly less prevalent in those in the over 61 age group than in the 21-30 year age group, and patients in the over 51 age group were less likely to have C-shaped root canals than those in other age groups. In previous studies [10, 15], C-shaped canals were likewise found to be less common in their 50s and 60s. It is our conjecture that as C-shaped canals are anatomically complex and more difficult for performing root canal treatment than other types of root canals, the incidence of extraction of teeth with C-shaped canals increases with age, resulting in a decreased prevalence with increasing age.

In this study, no difference was found in the frequency of C-shaped roots in the mandibular second molars according to tooth position, aligning with the results of earlier studies [10, 15]. However, women had a significantly higher prevalence of C-shaped roots than men, as supported by some previous Korean studies [16] but refuted by other studies [8, 10, 15]. These discrepancies may reflect differences in sample size and participants' ethnic background. In light of the present study results, clinicians should consider age, sex, and ethnicity when determining root canal morphology prior to root canal treatment.

Most of the C-shaped canals (75.3%) in the present study were bilateral, and similar results were reported in a previous study of a Chinese population [17]. Therefore, if a C-shaped canal is detected in a mandibular second molar, a C-shaped canal is also likely to be present in the contralateral second molar. The relatively large sample size of bilateral molar pairs in the present study enabled a more reliable analysis of the concurrency rate than was possible in other studies, which included a smaller number of bilateral pairs [10, 17].

The most common configuration of C-shaped canals was the C1 type (35.3%), as has been found in previous research [10, 15, 16]. The C1 type became less common as age increased, and the C3b type increased with age. As a rule, the canal and chamber volume are inversely proportional to age due to secondary dentin formation throughout life, which may eventually result in almost total pulp obliteration. In molar teeth, the floor of the chamber is the site of the greatest deposition [18]. These aging-related changes may have influenced the shift from the continuous C type to the separated C type.

In this study, 75.2% of teeth with C-shaped canals observed in CBCT appeared as fused roots on panoramic radiographs. Prior research has likewise pointed out that a fused root on radiographs of mandibular molars predicted the presence of a C-shaped anatomy. [14, 19, 20], and this suggestion appears to be confirmed by our findings. The middle wall thickness of the separated roots was 2.98 mm, which was thinner than that of the fused roots (4.15 mm). C-shaped root canals with thin middle walls can be observed as separated roots on panoramic radiographs. In C-shaped root canals that appeared as separated roots on panoramic radiographs, there was no significant difference in proportion between types; this finding indicates that the C3a type, which showed the lowest prevalence overall, occurred with a relatively high frequency in this context. Therefore, for C-shaped root canals that appear as separated roots on panoramic radiographs, it is likely that the middle wall is relatively thin and the possibility of the C3a type is relatively high.

The thickness of the lingual wall was significantly different between the orifice and 5 mm from the apex level in the middle zone for all types of C-shaped root canals. According to an earlier study, greater root canal wall thickness at the orifice was associated with a larger decrease in thickness towards the apical region, implying the need for particular care in teeth with thick root canal walls at the orifice region [9]. In the current study, the lingual wall was thickest at the orifice level in the middle zone, and the difference compared to the thickness at the apex was also the greatest. Therefore, particular caution may be needed to prevent strip perforation when preparing a root canal in this area. Preoperative canal wall thickness measurements could also be helpful for reducing the risk of strip perforation if previous CBCT images are available.

A limitation of this study is the distortion of CBCT images. Misalignment might distort the root geometry, leading to subtle errors in geometrical measurements, such as measurements of the wall thickness at each level. Artifacts related to misalignment are well-known issues [21]. These artifacts are particularly conspicuous for objects that are relatively far from the plane of the beam's rotation, which may be the case for mandibular second molars (as measured in this study) due to their relatively lateral location [9]. The possibility of distortions due to misalignment should be considered when interpreting these images and measurements.

The results of this study furnish useful information for C-shaped canal anatomy for effective endodontic treatment, serving as a valuable diagnostic tool in endodontics. However, CBCT cannot be taken routinely in all cases of nonsurgical endodontic treatment. Instead, CBCT must be used in endodontics only if the benefits to the patient outweigh the potential risks. However, retrospective root canal anatomy studies using existing CBCT scans can shed valuable light on ways to improve the success rate of both surgical and nonsurgical root canal treatment [22].

## 5. Conclusions

In this study, C-shaped canals were also observed at a high rate in mandibular second molars in Koreans than in other populations. The expression of C-shaped canals varies with age and sex, and the distribution of configuration types changes with age. The fused root shape that appears on panoramic radiography may be a predictor of C-shaped canals. The risk of strip perforation occurring in middle zone of the lingual wall during root canal preparation is the highest, and for this reason, more caution is required when performing nonsurgical or surgical endodontic procedures in the mandibular second molars.

## Figures and Tables

**Table 1:** Number and frequency of C-shaped root canals in mandibular molars by gender, age, and tooth position (*n* = 924).

	Gender			Tooth position			Age (years)						
	Male	Female	*P* value	Left	Right	*P* value	18–20	21–30	31–40	41–50	51–60	61–	*P* value
No. of teeth	350/1182	574/1326	<0.001	469/1254	455/1254	0.562	114/316	408/1014	136/340	124/314	72/242	70/282	<0.001
Prevalence (%)	29.6	43.3		37.4	36.3		36.1	40.2	40	39.5	29.8	24.8	

Distribution of sample ratios among each subgroups was analyzed using the chi-squared test. Significant different prevalence between male and female and among age subgroups were observed (*P* value < 0.001).

**Table 2:** Number and percentage of study subjects with C-shaped canals in the mandibular second molars by gender, unilateral, or bilateral status.

	Unilateral			
	Left, *n* (%)	Right, *n* (%)	Bilateral, *n* (%)	Total, *n* (%)
No. of patients				
Female (*n* = 663)	46 (6.9)	30 (4.5)	249 (37.6)	325 (49.0)
Male (*n* = 591)	26 (4.4)	28 (4.7)	148 (25.0)	202 (34.2)
Total no. of patients (*n* = 1254)	72 (5.7)	58 (4.6)	397 (31.7)	527 (42.0)

Data were expressed as the number and percentage. Distribution of sample ratios among each subgroups was analyzed using the chi-squared test and significantly different (*P* value < 0.001).

**Table 3:** The distribution of classification types according to age.

	Age	*N*, *n* (%)	C1, *n* (%)	C2, *n* (%)	C3a, *n* (%)	C3b, *n* (%)	c4, *n* (%)
	18–20	113 (100)	63 (55.8)	14 (12.4)	13 (11.5)	14 (12.4)	9 (8)
	21–30	402 (100)	153 (38.1)	86 (21.4)	72 (17.9)	77 (19.2)	14 (3.5)
	31–40	134 (100)	49 (36.6)	36 (26.9)	22 (16.4)	23 (17.2)	4 (3)
	41–50	124 (100)	31 (25)	33 (26.6)	24 (19.4)	34 (27.4)	2 (1.6)
	51–60	72 (100)	17 (23.6)	12 (16.7)	17 (23.6)	23 (31.9)	3 (4.2)
	61+	69 (100)	10 (14.5)	13 (18.8)	12 (17.4)	26 (37.7)	8 (11.6)
	Total	914 (100)	323 (35.3)	194 (21.2)	160 (17.5)	197 (21.6)	40 (4.4)
*P* value	18–20 vs. 21–30		0.001	0.045	0.140	0.127	0.075
	18–20 vs. 31–40		0.004	0.008	0.358	0.385	0.144
	18–20 vs. 41–50		<0.001	0.010	0.138	0.007	0.044
	18–20 vs. 51–60		<0.001	0.549	0.048	0.002	0.474
	18–20 vs. 61+		<0.001	0.331	0.369	<0.001	0.580
	21–30 vs. 31–40		0.837	0.234	0.793	0.701	1.000
	21–30 vs. 41–50		0.011	0.275	0.817	0.065	0.447
	21–30 vs. 51–60		0.026	0.451	0.329	0.022	1.000
	21–30 vs. 61+		<0.001	0.748	1.000	0.001	0.008
	31–40 vs. 41–50		0.061	1.000	0.651	0.067	0.751
	31–40 vs. 51–60		0.081	0.139	0.285	0.024	0.966
	31–40 vs. 61+		0.002	0.275	1.000	0.002	0.032
	41–50 vs. 51–60		0.964	0.156	0.600	0.610	0.533
	41–50 vs. 61+		0.127	0.299	0.886	0.189	0.008
	51–60 vs. 61+		0.245	0.907	0.481	0.590	0.184

To compare total sample ratios between age groups, the 2-sample proportion test was performed. A *P* value < 0.05 was considered to indicate statistical significance.

**Table 4:** Distribution of configuration types in separated versus fused roots on panoramic radiographs of teeth with C-shaped canal on CBCT.

Configuration type	Separated roots, *n* (%)	Fused roots, *n* (%)
C1	62 (25.6)	279 (38.2)
C2	56 (23.1)	153 (20.9)
C3a	65 (26.9)	121 (16.6)
C3b	59 (24.4)	178 (24.4)
Total	242	731
*P* value	0.863	<0.001

Data were expressed as number and percentage. Distribution of sample ratios among each subgroups was analyzed using the chi-square test. Distribution of configuration types in fused roots was significantly different (*P* value < 0.001).

**Table 5:** Mean values of the mesial, middle, and distal lingual wall thickness at the orifice and 5 mm from the apex by configuration type.

	Orifice	5 mm from apex	*P* value
Mesial zone thickness of the lingual wall			
C1	0.91 ± 0.33	0.95 ± 0.32	0.222
C2	1.04 ± 0.33	0.92 ± 0.32	0.463
C3a	0.73 ± 0.39	0.94 ± 0.33	0.365
C3b	1.41 ± 0.68	1.00 ± 0.33	0.036
Middle zone thickness of the lingual wall			
C1	2.23 ± 0.80	0.70 ± 0.28	<0.001
C2	2.60 ± 0.85	0.89 ± 0.41	<0.001
C3a	2.31 ± 0.66	0.70 ± 0.31	<0.001
Distal zone thickness of the lingual wall			
C1	1.44 ± 0.41	0.88 ± 0.31	<0.001
C2	1.30 ± 0.23	0.90 ± 0.27	0.038
C3a	1.46 ± 0.47	0.96 ± 0.34	0.042
C3b	1.12 ± 0.54	0.99 ± 0.35	0.274

Variables are presented as the mean ± standard deviation, and *P* values were computed using the Mann–Whitney *U* test. A *P* value < 0.05 was considered to indicate statistical significance.

## Data Availability

The data used to support the findings of this study are included within the article.
