# Erratum to “Prevalence and Morphology of C-Shaped Canals: A CBCT Analysis in a Korean Population”

**DOI:** 10.1155/2022/9841276

**Published:** 2022-02-12

**Authors:** Tae Yeon Lee, Sung Eun Yang, Kyung Jae Kim

**Affiliations:** ^1^Department of Conservative Dentistry, Yeouido St. Mary's Hospital, College of Medicine, The Catholic University of Korea, Seoul, Republic of Korea; ^2^Department of Conservative Dentistry, Seoul St. Mary's Dental Hospital, College of Medicine, The Catholic University of Korea, Seoul, Republic of Korea; ^3^Private Dental Clinic, Seoul, Republic of Korea

In the article titled “Prevalence and Morphology of C-Shaped Canals: A CBCT Analysis in a Korean Population” [1], the author order in the author list is stated incorrectly, and the corrected author order is shown above.
